# Intranasal fluticasone furoate in pediatric allergic rhinitis: randomized controlled study

**DOI:** 10.1038/s41390-020-01180-0

**Published:** 2020-10-02

**Authors:** Yamei Zhang, Ping Wei, Bobei Chen, Xiaoyan Li, Xianyang Luo, Xianming Chen, Mingliang Xiang, Lan Li, Sijun Zhao, Xuping Xiao, Xinmin Yang, Jie Chen, Yong Fu, Shuifang Xiao, Haixia Liu, Lei Cheng, Hongbing Yao

**Affiliations:** 1 Department of Otolaryngology, Head and Neck Surgery, Beijing Children’s Hospital, Capital Medical University, Beijing, China; 2 Department of Otolaryngology, The Children’s Hospital of Chongqing Medical University, Chongqing, China; 3 Department of Otolaryngology, The Second Affiliated Hospital, Wenzhou Medical University, Wenzhou, Zhejiang China; 4 Department of Otolaryngology, Head and Neck Surgery, Children’s Hospital Affiliated to Shanghai Jiao Tong University, Shanghai, China; 5 Department of Otolaryngology, Head and Neck Surgery, The First Affiliated Hospital, Medical College, Xiamen University, Xiamen, Fujian China; 6 Department of Otorhinolaryngology, Fuzhou General Hospital, Fuzhou, Fujian China; 7 Department of Otolaryngology, Head and Neck Surgery, Ruijin Hospital, Shanghai Jiao Tong University School of Medicine, Shanghai, China; 8 Department of Otolaryngology, Shenzhen Children’s Hospital, Shenzhen, Guangdong China; 9 Department of Otolaryngology, and Head and Neck Surgery, Hunan Children’s Hospital, Changsha, Hunan China; 10 Department of Otolaryngology, Head and Neck Surgery, Hunan Provincial People’s Hospital, The First Affiliated Hospital of Hunan Normal University, Changsha, Hunan China; 11 Department of Otolaryngology, Head and Neck Surgery, The Second Xiangya Hospital, Central South University, Changsha, Hunan China; 12 Department of Otorhinolaryngology, Head and Neck Surgery, Shanghai Children’s Medical Center, Shanghai Jiao Tong University School of Medicine, Shanghai, China; 13 Department of Otolaryngology, Head and Neck Surgery, The Children’s Hospital, Zhejiang University School of Medicine, Hangzhou, Zhejiang China; 14 Department of Otolaryngology, Head and Neck Surgery, Peking University First Hospital, Beijing, China; 15 Shanxi Children’s Hospital, Taiyuan, Shanxi China; 16 Department of Otorhinolaryngology & Clinical Allergy Center, The First Affiliated Hospital, Nanjing Medical University, Nanjing, Jiangsu China; 17 International Centre for Allergy Research, Nanjing Medical University, Nanjing, Jiangsu China

## Abstract

**Background:**

Intranasal corticosteroids are the most efficacious anti-inflammatory medications for allergic rhinitis (AR). However, the efficacy and safety of intranasal corticosteroids in children have not yet been subject to specific research in China. The aim of this study was to investigate the efficacy and safety of fluticasone furoate nasal spray (FFNS) in a Chinese pediatric population.

**Methods:**

In this phase 4 randomized, double-blind, placebo-controlled, multicenter study, pediatric AR patients aged 2–12 years were randomized 1:1:1, receiving either FFNS 55 µg or 110 µg or placebo. Electronic diary cards were completed to record symptoms, rescue medication use, and treatment compliance. Anterior rhinoscopy and overall response to therapy were evaluated and recorded.

**Results:**

Patients treated with FFNS at either dose experienced a significantly greater reduction in daily reflective total nasal symptom score compared with placebo. This was maintained in a younger subset of patients (2–6 years). Drug-related adverse events occurred in <20% of patients in all groups. FFNS was well tolerated at both doses.

**Conclusions:**

This study demonstrates favorable efficacy and safety profiles for FFNS 55 µg or 110 µg in Chinese pediatric populations (2–12 years), supporting its use in clinical treatment for AR children, including younger children aged 2–6 years.

**Impact:**

- The aim of this study was to investigate the efficacy and safety of intranasal fluticasone furoate in Chinese pediatric allergic rhinitis.
- This research not only addresses the deficiency in efficacy and safety data for intranasal corticosteroids in very young patients (aged 2–6 years) worldwide but also demonstrates that fluticasone furoate nasal spray shows a favorable benefit/risk profile at different dose levels.
- Our data will be of interest to the broad readership of *Pediatric Research* and will positively contribute to the dialog regarding the treatment of allergic rhinitis in children aged 2–6 years.

## Introduction

Allergic rhinitis (AR) is one of the most common allergies worldwide and is estimated to affect up to 40% of the global population.^1^ AR is common in children, and its prevalence in pediatric populations is increasing.^2^ AR is often classified based on the duration of symptoms as either intermittent (IAR: <4 days per week or lasting <4 weeks in duration) or persistent (PAR: ≥4 days per week and lasting ≥4 weeks in duration).^3^ Our previous study showed that the incidence of AR in Chinese subjects is rising.^4^ It is known to impact school performance and can affect a child’s integration with their peers, as well as causing anxiety and a degree of family dysfunction.^5–7^ Clinical trials are therefore necessary to find an effective treatment with a good safety profile for use in pediatric patients with AR.

Currently, intranasal corticosteroids are considered to be the most effective medication for controlling the symptoms of AR.^8^ One such medication is fluticasone furoate nasal spray (FFNS; AVAMYS^©^, GlaxoSmithKline plc.), a once-daily intranasal corticosteroid that has demonstrated therapeutic efficacy and safety for the treatment of AR in a Chinese population study in 2012.^9^ The approved prescribing guidelines in China recommend a first dose of FFNS of 110 µg once daily for adults and adolescents (≥12 years). However, FFNS is not in general clinical use in Chinese children due to a lack of efficacy and safety data.^10^

The purpose of this study was to investigate the efficacy and safety of FFNS compared with placebo in a Chinese pediatric population aged 2–12 years. In addition, we also report the results of a post hoc subgroup analysis that investigated age-related comparisons of AR classification, treatment response and safety, and nasal and ocular symptom severity within this study population. This is a study population which we believe is the youngest included in an intranasal corticosteroid study in China to date.

## Materials and methods

### Study design and patients

The phase 4 randomized, double-blind, placebo-controlled, multicenter study was conducted at 16 centers in China (ClinicalTrials.gov NCT02424539). The trial was approved by the independent ethics committee of each research center and was conducted in accordance with the Declaration of Helsinki and GCP guidelines. The parents or guardians (carers) of all patients provided written, informed consent ahead of study initiation. Additionally, patients who were able to read and understand the study information signed an Informed Acceptance Form.

**Fig. 1:**
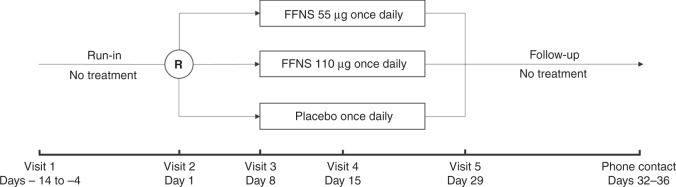
Study design. FFNS fluticasone furoate nasal spray, R randomization.

This study evaluated the efficacy and safety of once-daily FFNS 55 μg and 110 μg versus vehicle placebo nasal spray in pediatric patients with AR. The study had a treatment-free run-in period (4–14 days) before randomization, followed by 4 weeks of double-blind treatment and a 3–7-day treatment-free follow-up period (Fig. 1).

Patients were eligible for inclusion into the study if they met criteria for either IAR or PAR according to Chinese guidelines,^11^ and were 2–12 years of age. Exclusion criteria included comorbid disorders that may have affected study results (including nasal polyps, eye, or upper respiratory infection within 2 weeks of the start of the screening period, asthma [except mild intermittent cases], rhinitis medicamentosa, vasomotor AR, or eosinophil rhinitis), recent nasal septal surgery or perforation, and traveling for >48 h during the study (potentially experiencing a change in allergens). Use of medication that could significantly affect the course of AR or interact with the study drug was not permitted.

In addition, patients had to meet the following randomization criteria: nasal and/or ocular symptoms on the morning of randomization; an average reflective total nasal symptom score (rTNSS) of ≥6 for the last 8 assessments (4 assessments in the morning and 4 assessments in the afternoon) before randomization, including the morning assessment at randomization; and average reflective nasal score symptom assessment for congestion of ≥2 for the last 8 assessments (4 assessments in the morning and 4 assessments in the afternoon) before randomization, including the morning assessment at randomization.

### Randomization and masking

Patients who met eligibility and randomization criteria were stratified by age (≥2 to ≤6 or >6 to ≤12 years) and AR classification (IAR or PAR), then randomly allocated in a 1:1:1 ratio to receive once-daily FFNS 55 μg, FFNS 110 μg, or placebo nasal spray. The randomization schedule was generated by Clinical Statistics, using validated internal software. Treatment interventions were masked to patients, carers, investigators, and treating physicians.

### Procedures

Eligible patients received once-daily FFNS 55 μg, FFNS 110 μg, or placebo for 4 weeks. These doses are approved for treatment in China and are in keeping with global phase III studies previously carried out in pediatric cohorts.^12–15^ Patients and carers were given two blinded treatment kits. Each kit contained a nasal spray device, and based on treatment allocation, patients received either FFNS 55 μg in both kits or one kit with FFNS 55 μg and one kit with placebo. One nasal spray device was labeled “Device A” and the other “Device B” Patients or their carers were instructed to administer one spray from Device A into each nostril, followed by one spray from Device B into each nostril once daily in the morning. Loratadine syrup was provided as rescue medication for use as needed during the 4-week treatment period.

Electronic diary cards completed daily during the 4-week treatment period were used to record carer-reported symptom assessments, rescue loratadine use, and treatment compliance. Efficacy measures of subjective symptoms were based on categorical scale ratings provided by patients’ carers throughout the study. Anterior rhinoscopic findings were evaluated by investigators using a categorical scale at baseline (Visit 2) and at each subsequent study visit (Visits 3, 4, 5, or early withdrawal). Overall response to therapy was evaluated using a 1–7 scale (1 = significantly improved; 7 = significantly worse) after 2 and 4 weeks of treatment (or at early discontinuation) by patients or their carers. These responses were analyzed using logistic regression. Safety was assessed by monitoring adverse events, assessing vital signs, electrocardiographs, and clinical laboratory tests and by carrying out physical and nasal examinations.

### Outcomes

The primary comparisons of interest between treatment groups were for FFNS 55 μg or 110 μg versus placebo. The primary endpoint was the mean change from baseline in daily rTNSS over the first 2 weeks of treatment. Secondary endpoints included overall response to therapy, mean change from baseline in daily rTNSS, reflective total ocular symptom score (rTOSS) over 4 weeks, intranasal finding score by anterior rhinoscopy, rescue loratadine use, and safety and tolerability. For the post hoc analysis, age (≥2 to ≤6 or >6 to ≤12 years) and severity (moderate and severe) of nasal and ocular symptoms were used as parameters to evaluate the efficacy and safety of the 2 FFNS treatment groups.

### Statistical analysis

A sample size of 360 randomized patients (120 per treatment group) was planned in order to ensure 100 eligible patients per group and at least 50 patients who were 2–6 years of age, assuming that 17% of patients would not be evaluable for the primary analysis. Based on a sample size of 100 patients per arm, the probability of observing a positive trend for the primary endpoint was 95% for patients aged 2–12 years, 90% for patients aged 2–6 years, and 88% for both age groups.

The intent-to-treat (ITT) population was used for efficacy analyses and included all randomized patients who received at least one dose of study medication. A planned subgroup analysis of the ITT population comprising only patients aged 2–6 years was performed to investigate the efficacy and safety of FFNS in younger patients. Patients from the ITT population were included in the analysis of two subgroups: children aged 2–6 years and 6–12 years.

Mean change from baseline in daily rTNSS/rTOSS by age group were analyzed using analysis of covariance (ANCOVA) for baseline daily rTNSS, classification of AR (IAR or PAR), age (continuous), sex, and treatment. Based on the subset of the ITT population with moderate and severe total ocular symptoms at baseline, mean change from baseline over the weeks in daily rTNSS/rTOSS was also analyzed by baseline severity. For mean change from baseline in daily rTOSS, both the overall population and the age groups were analyzed based on the subset of ITT population with moderate and severe total ocular symptoms at baseline.

The per-protocol population included all patients in the ITT population who did not have any full protocol deviations.

## Results

This study was conducted from September 2015 to October 2017. Overall, 505 patients were screened and 358 patients were randomized. Two patients who failed screening and were randomized in error were not included in the study population. All randomized patients received at least one dose of intended treatment and comprised the ITT population: FFNS 55 µg once daily (*n* = 119), FFNS 110 µg once daily (*n* = 119), and placebo group (*n* = 120). The end of the study was defined as the last patient’s last visit at follow-up.

### Baseline characteristics

Baseline characteristics were well balanced across treatment arms (Table 1). The percentage of patients aged 2 and 12 years were the lowest of all the ages within the ITT population (3%) (full details of the number and percentages of patients by age in the ITT population can be found in Supplementary Table S1). Nocturnal nasal symptoms were higher than daytime symptoms (overall mean [standard deviation] symptom scores were 8.6 [1.48] at nighttime and 8.3 [1.45] in the daytime). A much higher percentage of patients had PAR (92%) than IAR (8%), and there were a greater proportion of males across all groups (*n* = 246; 69%). All patients were of Asian or East Asian race.

**Table 1:** Baseline characteristics.

Characteristic	FFNS 55 µg once daily (*n* = 119)	FFNS 110 µg once daily (*n* = 119)	Placebo (*n* = 120)	Total (*N* = 358)	Characteristic	FFNS 55 µg once daily (*n* = 119)	FFNS 110 µg once daily (*n* = 119)	Placebo (*n* = 120)	Total (*N* = 358)
Age, years					Ocular symptoms severity at baseline^b^				
Mean (SD)	6.9 (2.54)	6.6 (2.54)	6.8 (2.64)	6.8 (2.57)	All patients, *n* (%)^b^	119	119	120	358
Age group, *n* (%)^a^					None	5 (4)	9 (8)	6 (5)	20 (6)
2 to ≤6 years	59 (50)	60 (50)	60 (50)	179 (50)	Mild	60 (50)	57 (48)	45 (38)	162 (45)
6 to ≤12 years	60 (50)	59 (50)	60 (50)	179 (50)	Moderate	48 (40)	43 (36)	55 (46)	146 (41)
Sex, *n* (%)^a^					Severe	6 (5)	10 (8)	14 (12)	30 (8)
Female	38 (32)	32 (27)	42 (35)	112 (31)	Mean (SD)	2.9 (1.92)	3.1 (2.09)	3.4 (1.94)	3.1 (1.99)
Male	81 (68)	87 (73)	78 (65)	246 (69)	Median	2.9	3.0	3.4	3.0
Asian/East Asian race, *n*^a^	119	119	120	358	Min–max	0.0–8.4	0.0–8.0	0.0–8.1	0.0–8.4
Nasal symptoms severity at baseline^b^					≥2 to ≤6 years, *n* (%)^b^	59	60	60	179
All patients, *n* (%)^b^	119	119	120	358	None	4 (7)	4 (7)	4 (7)	12 (7)
Moderate	53 (45)	52 (44)	49 (41)	154 (43)	Mild	30 (51)	31 (52)	22 (37)	83 (46)
Severe	66 (55)	67 (56)	71 (59)	204 (57)	Moderate	22 (37)	21 (35)	27 (45)	70 (39)
Mean (SD)	8.4 (1.46)	8.3 (1.25)	8.5 (1.44)	8.4 (1.38)	Severe	3 (5)	4 (7)	7 (12)	14 (8)
Median	8.3	8.3	8.4	8.3	Mean (SD)	2.9 (1.93)	3.1 (1.91)	3.3 (1.89)	3.1 (1.91)
Min–max	6.0–12.0	6.0–11.9	6.0–12.0	6.0–12.0	Median	2.8	3.0	3.3	3.0
≥2 to ≤6 years, *n* (%)^b^	59	60	60	179	Min–max	0.0–7.5	0.0–6.9	0.0–7.1	0.0–7.5
Moderate	28 (47)	29 (48)	31 (52)	88 (49)	>6 to ≤12 years, *n* (%)^b^	60	59	60	179
Severe	31 (53)	31 (52)	29 (48)	91 (51)	None	1 (2)	5 (8)	2 (3)	8 (4)
Mean (SD)	8.3 (1.49)	8.2 (1.27)	8.4 (1.58)	8.3 (1.45)	Mild	30 (50)	26 (44)	23 (38)	79 (44)
Median	8.1	8.1	8.0	8.1	Moderate	26 (43)	22 (37)	28 (47)	76 (42)
Min–max	6.0–12.0	6.0–11.9	6.0–12.0	6.0–12.0	Severe	3 (5)	6 (10)	7 (12)	16 (9)
>6 to ≤12 years, *n* (%)^b^	60	59	60	179	Mean (SD)	3.0 (1.91)	3.1 (2.28)	3.5 (2.01)	3.2 (2.07)
Moderate	25 (42)	23 (39)	18 (30)	66 (37)	Median	3.0	3.0	3.5	3.1
Severe	35 (58)	36 (61)	42 (70)	113 (63)	Min–max	0.0–8.4	0.0–8.0	0.0–8.1	0.0–8.4
Mean (SD)	8.6 (1.43)	8.5 (1.21)	8.6 (1.27)	8.6 (1.30)	Allergic rhinitis type, *n* (%)^b^				
Median	8.3	8.4	8.6	8.4	Intermittent	11 (9)	9 (8)	7 (6)	27 (8)
Min–max	6.1–12.0	6.0–11.1	6.0–12.0	6.0–12.0	Persistent	108 (91)	110 (92)	113 (94)	331 (92)

*FFNS* fluticasone furoate nasal spray, *SD* standard deviation.

^a^Primary analysis data.

^b^Post hoc analysis data.

Subgroup data analysis showed that the severity of nasal symptom status at baseline was similar between treatment groups in the ITT population; however, the 6–12-year age group had higher mean scores for baseline rTNSS than the 2–6-year group. The percentage of patients with moderate and severe baseline rTNSS were similar in the 2–6-year age group, but for the 6–12-year age group, there were a higher percentage of patients with a severe baseline compared with the moderate baseline rTNSS (Table 1).

The severity of individual nasal symptoms at baseline in the ITT population was also analyzed post hoc. Overall, severe nasal congestion was experienced by a higher percentage of patients (74%) compared with runny nose (43%), itchy nose (42%), and sneezing (34%) (Supplementary Table S2). When the ITT population was stratified by age group, a higher percentage of patients reported severe baseline symptoms in the 6–12-year age group compared with the 2–6-year age group, specifically: nasal congestion (80% versus 68%), runny nose (52% versus 34%), and sneezing (37% versus 30%).

At baseline, 94% of patients demonstrated ocular symptoms. Post hoc analysis of ocular symptom severity demonstrated a lower percentage in the severe baseline rTOSS of the ITT population compared with the mild and moderate baseline rTOSS: 8% versus 45% and 41%, respectively. For the ITT population by age group, the percentages of patients with mild, moderate, and severe baseline rTOSS were similar: 46, 39, and 8% for the 2–6-year age group, respectively, and 44, 42, and 9% for the 6–12-year age group, respectively (Table 1). Overall, eye itching/burning had the highest mean score (1.3) among the three individual ocular symptoms (Supplementary Table S2).

In the post hoc analysis of the severity of baseline intranasal finding score by anterior rhinoscopy in the ITT population, the proportion of patients scored as severe was higher when evaluated by physicians than when evaluated by patients’ self-reported results: 80% compared with 57%, respectively.

### Protocol deviations

Overall, the incidence of important protocol deviations was similar among the three treatment groups in this study (Supplementary Table S3). The most common important protocol deviations were excluded medication (41 subjects), wrong study treatment (30 subjects), and eligibility criteria not met (21 subjects), which occurred from 6 to 11% of subjects across the groups.

### Efficacy outcomes

In total, 92% of patients completed the study. In the placebo group, 12% of patients withdrew early, compared with 7% in both the once-daily FFNS 55 µg group and the once-daily FFNS 110 µg group, respectively (Consort diagram: Supplementary Fig. S1). The leading reason for early withdrawal was reaching protocol-defined stopping criteria.

### Mean change from baseline in daily rTNSS over first 2 weeks (primary analysis, primary endpoint)

**Fig. 2:**
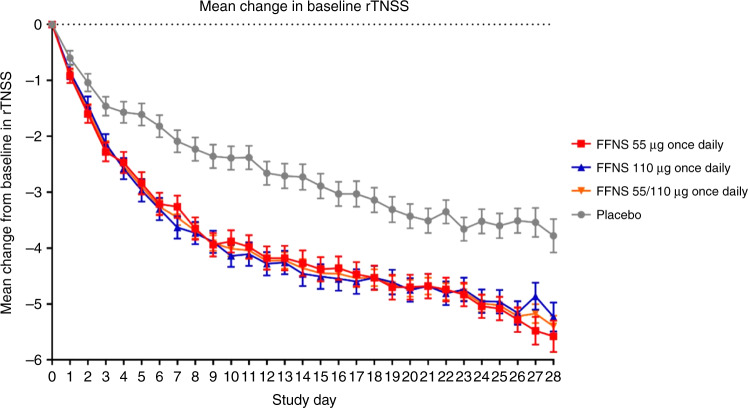
Mean change from baseline in daily rTNSS (ITT population). FFNS fluticasone furoate nasal spray, ITT intent to treat, rTNSS reflective total nasal symptom scores. Data are presented for the entire treatment period and were statistically significant (*P* < 0.001) over 2 and 4 weeks for all dose variations when compared with placebo.

The primary endpoint of this study was the mean change from baseline in daily rTNSS over the first 2 weeks of treatment (Table 2 and Fig. 2). Baseline mean daily rTNSS was similar across treatment groups, but patients treated with FFNS 55 or 110 µg experienced a significantly greater (*P* < 0.001) reduction in daily rTNSS compared with placebo over the first 2 weeks, with least-squares (LS) mean differences of −1.23 and −1.32, respectively; indeed, both treatment groups demonstrated significant differences from day 3 compared to the placebo group (Fig. 2).

**Table 2:** Mean change from baseline in daily rTNSS over the first 2 and 4 weeks of treatment (ITT population).

Daily rTNSS	All patients^c^				≥2 to ≤6 years^d^				>6 to ≤12 years^d^			
	FFNS 55 µg once daily (*n* = 119)	FFNS 110 µg once daily (*n* = 119)	FFNS 55/110 µg once daily (*n* = 238)	Placebo (*n* = 120)	FFNS 55 µg once daily (*n* = 59)	FFNS 110 µg once daily (*n* = 60)	FFNS 55/110 µg once daily (*n* = 119)	Placebo (*n* = 60)	FFNS 55 µg once daily (*n* = 60)	FFNS 110 µg once daily (*n* = 59)	FFNS 55/110 µg once daily (*n* = 119)	Placebo (*n* = 60)
Baseline mean (SD)	8.4 (1.46)	8.3 (1.25)	8.4 (1.36)	8.5 (1.44)								
Week 1–2 mean (SD)	5.2 (1.94)	5.1 (2.10)	5.2 (2.02)	6.5 (1.72)								
Week 1–4 mean (SD)	4.5 (1.94)	4.4 (2.08)	4.4 (2.01)	5.9 (1.79)								
Week 1–2 change from baseline												
Mean change (SD)	−3.2 (1.95)	−3.2 (1.99)	−3.2 (1.96)	−2.0 (1.58)								
LS mean change (SE)^a^	−3.18 (0.16)	−3.28 (0.16)	−3.23 (0.11)	−1.95 (0.16)	−3.06 (0.22)	−3.35 (0.22)	−3.21 (0.16)	−2.04 (0.22)	−3.30 (0.24)	−3.19 (0.24)	−3.25 (0.17)	−1.87 (0.24)
LS mean difference^a^	−1.23	−1.32	−1.28		−1.03	−1.32	−1.17		−1.43	−1.32	−1.37	
*P* value versus placebo^a^	**	**	**		**	**	**		**	**	**	
95% CI^a^	(−1.68, −0.78)	(−1.77, −0.87)	(−1.66, −0.89)		(−1.65, −0.41)	(−1.93, −0.70)	(−1.71, −0.64)		(−2.09, −0.77)	(−1.98, −0.65)	(−1.95, −0.80)	
*P* value versus FF 55 µg						0.357				0.729		
95% CI^b^						(−0.91, 0.33)				(−0.55, 0.78)		
Week 1–4 change from baseline												
Mean change (SD)	−3.9 (1.99)	−4.0 (2.05)	−4.0 (2.02)	−2.6 (1.80)								
LS mean change (SE)^a^	−3.93 (0.17)	−4.03 (0.17)	−3.98 (0.12)	−2.57 (0.17)	−3.90 (0.23)	−4.17 (0.22)	−4.03 (0.16)	−2.71 (0.22)	−3.96 (0.25)	−3.88 (0.25)	−3.92 (0.18)	−2.44 (0.25)
LS mean difference^a^	−1.36	−1.46	−1.41		−1.19	−1.46	−1.33		−1.52	−1.44	−1.48	
*P* value versus placebo^a^	**	**	**		**	**	**		**	**	**	
95% CI^a^	(−1.83, −0.90)	(−1.92, −0.99)	(−1.81, −1.01)		(−1.82, −0.56)	(−2.09, −0.84)	(−1.87, −0.78)		(−2.21, −0.83)	(−2.14, −0.74)	(−2.08, −0.88)	
*P* value versus FF 55 µg						0.396				0.816		
95% CI^b^						(−0.09, 0.36)				(−0.62, 0.78)		

*CI* confidence interval, *FFNS* fluticasone furoate nasal spray, *ITT* intent to treat, *LS* least squares, *LS mean difference* LS mean change in active treatment minus LS mean change in placebo, *rTNSS* reflective total nasal symptom scores, *SD* standard deviation, *SE* standard error.

**P < 0.001 compared with placebo.

^a^The baseline period for diary card endpoints was defined as 4 days before randomization, including the morning symptom assessment on the randomization (i.e., treatment initiation) date.

^b^Based on ANCOVA adjusting for baseline daily rTNSS, classification of allergic rhinitis (intermittent or persistent), age (as continuous variable), sex, and treatment.

^c^Primary analysis data.

^d^Post hoc analysis data.

A significant difference was also observed for the pooled FFNS 55/110 µg group, with an LS mean difference of −1.28 versus placebo (*P* < 0.001). Similar trends for significant improvements versus placebo with FFNS 55, 110, and 55/110 µg were also observed in the younger subset of ITT patients aged 2–6 years.

In both the ITT population and in the subset of patients from the ITT population aged 2–6 years, the trend for significant improvements with FFNS 55, 110, and 55/110 µg over placebo was maintained when examining the mean change in daily rTNSS from baseline over the whole 4 weeks of the study (Table 2) and also on a week-by-week basis.

### Mean daily rTNSS over each week

Overall, in the ITT population, data showed that the LS mean changes from baseline in rTNSS were numerically higher for FFNS 55, 110, and pooled FFNS 55/110 µg versus placebo. The LS mean difference was statistically significant (*P* < 0.001) over the first 2 and 4 weeks.

Post hoc analyses demonstrated that there was no statistical significance in either age group between treatment with FFNS 55 µg and FFNS 110 µg. Children with moderate and severe baseline nasal symptoms showed the same statistically significant LS mean changes from baseline in rTNSS as the ITT population versus placebo (*P* < 0.001). There was no statistical significance in the LS mean changes from baseline in rTNSS between the FFNS 55 µg group and the FFNS 110 µg group when patients with moderate or severe baseline severity were compared over the first 2 weeks (moderate, *P* = 0.988; severe, *P* = 0.639) and 4 weeks (moderate, *P* = 0.930; severe, *P* = 0.635). Although not statistically significant, numerically higher LS mean changes from baseline in rTNSS were observed in the FFNS 110 µg group compared with the FFNS 55 µg group in patients with severe baseline symptoms: −1.48 versus −1.32, respectively, over 2 weeks, and −1.61 versus −1.45, respectively, over 4 weeks.

### Mean change from baseline in daily rTOSS (primary analysis, secondary endpoint)

In the first 2 weeks of the study, mean daily rTOSS was slightly higher for patients treated with placebo (2.1), compared with those treated with FFNS 55 µg (1.8), FFNS 110 µg (1.7), and the pooled FFNS 55/110 µg group (1.7). The LS mean changes from baseline in the experimental groups were not statistically significant when compared with placebo for this timeframe; similar results were observed during the post hoc analysis in both the 2–6-year and the 6–12-year age subgroups (Supplementary Table S4).

Examining the mean daily rTOSS over 4 weeks of treatment, patients in the placebo group again had a slightly higher mean score (1.9) compared with the FFNS 55 µg (1.5), FFNS 110 µg (1.4), and pooled FFNS 55/110 µg (1.5) groups. Over this timeframe, however, FFNS 110 µg demonstrated a significant LS mean difference in change from baseline when compared with placebo (*P* = 0.035; Supplementary Table S4). No significant differences were observed between the experimental groups and placebo in the subset of patients aged 2–6 years, irrespective of the time period.

Post hoc analyses demonstrated that the LS mean changes from baseline in rTOSS in patients with moderate baseline ocular symptoms were observed to be statistically significant between the FFNS 55 µg group and the FFNS 110 µg group over the first 4 weeks (−0.06 versus −0.58, *P* = 0.046). There was no statistical significance in the LS mean changes from baseline in rTOSS between the FFNS 110 µg group compared with the FFNS 55 µg group in patients with severe baseline ocular symptoms, possibly due to the small sample size: placebo *N* = 14, FFNS 55 µg *N* = 6, FFNS 110 µg *N* = 10.

### Mean change from baseline of intranasal finding score by anterior rhinoscopy (primary analysis, secondary endpoint)

The mean intranasal finding score at baseline was similar across treatment groups in the ITT population, ranging from 9.6 to 9.7. After both 2 and 4 weeks of treatment, the intranasal finding scores with FFNS 55 µg and FFNS 110 µg were significantly reduced from baseline versus placebo (*P* < 0.001; Supplementary Table S5). Notably, this trend was also observed in the ITT subset of patients aged 2–6 years.

### Rescue medication, overall response (primary analysis, secondary endpoint)

Use of rescue medication was very low across all treatment groups for the ITT population. In the first 2 weeks, the mean number of days without the use of rescue medication for patients treated with FFNS 55 µg and 110 µg was 13.3 and 13.4, respectively. This was slightly higher than for patients treated with placebo (12.8 days). When comparing all experimental treatments versus placebo, the LS mean differences were all significant: FFNS 55 µg, *P* = 0.048; FFNS 110 µg, *P* = 0.011; FFNS 55/110 µg, *P* = 0.009. These statistically significant LS mean differences were maintained for all experimental groups over the 4-week study period.

In the primary analysis of the subset of patients aged 2–6 years, while the mean number of days without the use of rescue medication in the first 2 weeks was slightly higher for treatment groups versus placebo (FFNS 55 µg, 13.3; FFNS 110 µg, 13.5; placebo, 12.9), statistically significant LS mean differences were not observed in this time period. However, over 4 weeks of treatment, statistically significant LS mean differences were observed for all treatment groups compared with placebo (FFNS 55 µg, *P* = 0.007; FFNS 110 µg, *P* < 0.001; FFNS 55/110 µg, *P* < 0.001).

After the first 2 weeks of treatment, 33% of patients treated with FFNS 55 µg and 43% of patients treated with FFNS 110 µg rated their overall response to therapy as “significantly improved,” compared with only 12% of patients in the placebo group (*P* < 0.001). A similar trend was observed in the subset of patients aged 2–6 years: significantly more patients treated with FFNS 55 µg (*P* = 0.005) and FFNS 110 µg (*P* < 0.001) had their overall response to treatment rated by their carers as “significantly improved,” compared with those treated with placebo. This trend was also maintained after 4 weeks of treatment.

### Safety outcomes

Overall, both FFNS 55 µg and FFNS 110 µg were well tolerated. Exposure was similar across all treatment groups in both the ITT population and the subset of patients aged 2–6 years. In the ITT population, the mean number of days that patients were exposed to treatment was 27.2 for placebo, compared with 27.9 and 28.9 for FFNS 55 μg and 110 µg, respectively. Most patients in the ITT population in all treatment groups remained on treatment for over 28 days.

In the ITT population, the incidence of adverse events was comparable between both the placebo and FFNS 55 µg groups (43% versus 44%, respectively) but was numerically higher in the FFNS 110 µg group (55%). The most common adverse events with an incidence of ≥3% that occurred at a higher rate in the treatment groups than in the placebo group were upper respiratory tract infection, cough, and sinusitis (Table 3).

**Table 3:** Incidence of adverse events and drug-related adverse events, overall and by age subgroup (ITT population).

Adverse events	Proportion of patients, %		
	FFNS 55 µg once daily	FFNS 110 µg once daily	Placebo
All patients^a^, *n*	119	119	120
Patients with any adverse event^b^	44	55	43
Upper respiratory tract infection	22	16	13
Cough	2	7	4
Sinusitis	3	< 1	< 1
Any drug-related adverse event^c^	14	19	19
Epistaxis	5	8	12
Cough	2	3	< 1
Sinusitis	3	< 1	< 1
≥2 to ≤6 years^d^, *n*	59	60	60
Patients with any adverse event	46	65	53
Patients with any drug-related adverse event	19	17	23
>6 to ≤12 years,^d^ *n*	60	59	60
Patients with any adverse event	42	46	35
Patients with any drug-related adverse event	10	22	15

*FFNS* fluticasone furoate nasal spray, *ITT* intent to treat.

^a^Primary analysis data.

^b^Occurring in ≥3% of patients in any treatment group and more common than placebo.

^c^Occurring in ≥2% of patients in an experimental group.

^d^Post hoc analysis data.

Similar trends were reported in the primary analysis for the younger subset of patients aged 2–6 years. Again, FFNS 110 µg was associated with a slightly higher rate of adverse events (65% versus 46% [FFNS 55 µg] and 53% [placebo]), and the most common adverse event was upper respiratory tract infection. In the post hoc analysis of the ITT population by age group, the 6–12-year age subset had a lower incidence of adverse events in all treatment groups.

Drug-related adverse events occurred in <20% of patients in all treatment groups. The most common drug-related adverse event in all groups was epistaxis; this was also the most common drug-related adverse event in the subset of patients aged 2–6 years, occurring in 15% of patients treated with placebo compared with 5% and 7% of patients treated with FFNS 55 µg and 110 µg, respectively. All epistaxis events were mild in intensity, with the exception of one patient in the placebo group in whom the event was unresolved. The 6–12-year age subset had a lower incidence of drug-related adverse events in the placebo and FFNS 55 µg treatment groups (post hoc analysis).

Only one serious adverse event of tonsillitis was reported in a 4-year-old patient treated with FFNS 110 µg but was not considered to be related to the study drug. The patient was withdrawn from the study due to the event.

## Discussion

The primary objective of this study was to further establish the efficacy and safety of once-daily FFNS 55 µg or 110 µg in Chinese pediatric patients aged 2–12 years, with additional subgroup analysis of the 2–6-year age group.

This study met its primary efficacy endpoint: a statistically significant greater treatment effect was observed in the mean change from baseline in daily rTNSS compared with FFNS 55 µg and 110 µg compared with placebo, in both the ITT population and the subset of patients aged 2–6 years, over 2 and 4 weeks of treatment. FFNS treatment at both doses also demonstrated efficacy in managing other AR symptoms within the ITT population. Over 4 weeks, FFNS 110 µg demonstrated a significantly greater reduction in daily rTOSS compared with placebo, and a significant reduction in anterior rhinoscopic findings (assessing the nasal mucosa, secretory volume, and description of rhinorrhea) was associated with FFNS irrespective of dose throughout the treatment period. The observed increase in the number of days without use of loratadine rescue medication for patients on active FFNS treatment also reflects the reduction in symptoms achieved in the ITT population. Generally, results were similar between the ITT population and the pre-specified younger subgroup of patients aged 2–6 years.

FFNS receptor binding studies showed a higher affinity for glucocorticoid receptors compared with other corticosteroids and, in addition, greater potency in attenuating inflammatory state.^15^ In our study, the 110 µg once-daily dose of FFNS consistently demonstrated numerically greater reductions from baseline in patients with severe symptoms compared with the FFNS 55 µg once daily, although this reduction was not statistically significant. Glucocorticoids reduce inflammation through a combination of glucocorticoid receptors. The possible explanation for the similar change in rTNSS seen across both the 55 µg and 110 μg treatment groups may be that the glucocorticoid receptors in the nasal mucosa have reached saturation point at the low dose (≤55 μg) according to the dose–response relationship of the glucocorticoid.^16^

The safety findings of the present study show that both doses of FFNS were well tolerated in children aged 2–12 years. The most common drug-related adverse event in all groups was epistaxis, and all epistaxis events were mild in intensity, occurring in 15% of patients treated with placebo compared with 5 and 7% of patients treated with FFNS 55 µg and 110 µg, respectively. Low-dose FFNS has a lower incidence of drug-related adverse events. It is important that AR therapy for pediatric patients should not compound the problems that AR can cause. Considering that children with poorly controlled AR often struggle with diminished alertness, inability to concentrate, and irritability at school,^4^ it is highly likely that medications linked to drowsiness could ultimately exacerbate these issues. In our study, there were no new safety signals.

Our efficacy and safety findings therefore support the use of FFNS in Chinese children, building on previous findings in Chinese adults and adolescents (≥12 years).^8^ The data presented here would suggest treating Chinese patients between the ages of 2 and 12 with an initial once-daily dose of 55 µg FFNS. In cases which are initially non-responsive, the dose could then be increased up to a maximum of 110 µg — once symptoms are under control, the dose could be reduced to 55 µg. While we did not give particular focus to the efficacy of these doses at different disease severities, this is research that may be valuable for future studies to consider.

FFNS is a once-daily treatment for AR, unlike alternative intranasal corticosteroid sprays that require multiple doses throughout the day.^8^ This could be important as both dosing and treatment regimen have been cited as factors that could affect adherence.^17^ For pediatric patients and their carers, the once-daily dosing requirement for FFNS may be considered more convenient and could therefore positively influence treatment adherence and ultimately lead to improved clinical outcomes.

However, there also lies a potential limitation of this study: it is possible that the carer recording symptoms in the electronic diary on the patient’s behalf might have over- or under-recorded AR symptoms—possibly introducing bias. Equally, the observed discrepancy between the number of patients having a physician-measured severe score for nasal symptoms, compared with using patient self-test results, may also have been subject to bias. According to the IAR/PAR classification of the children enrolled in this study, PAR accounted for 92%, which is much higher than in previous studies.^18^ Patients were recruited from 16 centers across China: 3 sites in northern China from which only 13 patients were enrolled, and 13 sites in Southern China. IAR is known to be common in the northern regions of China than PAR^1,10^ therefore, it is likely that site bias existed, which may have accounted for the particularly high proportion of PAR in the study.

In conclusion, the results of this study suggest that once-daily FFNS is an effective treatment with a reassuring safety profile in children aged 2–12 years, thus affirming its suitability for treating Chinese children with AR.

## Supplementary information

Supplementary Material

## Data Availability

Anonymized individual participant data and study documents can be requested for further research from www.clinicalstudydatarequest.com.
