# Supplementary material for: Intranasal fluticasone furoate in pediatric allergic rhinitis: randomized controlled study
Source: Pediatr Res. 2020 Oct 2;89(7):1832–9. doi: 10.1038/s41390-020-01180-0 (PMC8249237; doi:10.1038/s41390-020-01180-0)
Supplement: Supplementary file 1 — Supplementary Material [file 41390_2020_1180_MOESM1_ESM.docx]

SUPPLEMENTARY MATERIAL

**Intranasal fluticasone furoate in pediatric allergic rhinitis: randomized controlled study** Yamei Zhang^1^, Ping Wei^2^, Bobei Chen^3^, Xiaoyan Li^4^, Xianyang Luo^5^, Xianming Chen^6^, Mingliang Xiang^7^, Lan Li^8^, Sijun Zhao^9^, Xuping Xiao^10^, Xinmin Yang^11^, Jie Chen^12^, Yong Fu^13^, Shuifang Xiao^14^, Haixia Liu^15^, Lei Cheng^16,17^, Hongbing Yao^2*^

^1^Department of Otolaryngology, Head and Neck Surgery, Beijing Children’s Hospital, Capital Medical University, Beijing, China

^2^Department of Otolaryngology, Children’s Hospital of Chongqing Medical University, Chongqing, China

^3^Department of Otolaryngology, The Second Affiliated Hospital, Wenzhou Medical University, Wenzhou, Zhejiang, China

^4^Department of Otolaryngology, Head and Neck Surgery, Children’s Hospital Affiliated to Shanghai Jiaotong University, Shanghai, China

^5^Department of Otolaryngology, Head and Neck Surgery, The First Affiliated Hospital, Medical College, Xiamen University, Xiamen, Fujan, China

^6^Department of Otorhinolaryngology, Fuzhou General Hospital, Fuzhou, China

^7^Department of Otolaryngology, Head and Neck Surgery, Ruijin Hospital, Shanghai Jiao Tong University School of Medicine, Shanghai, China

^8^Department of Otolaryngology, Shenzhen Children’s Hospital, Shenzhen, Guangdong, China

^9^Department of Otolaryngology, Head and Neck Surgery, Hunan Children’s Hospital, Changsha City, Hunan, China

^10^Department of Otolaryngology, Head and Neck Surgery, Hunan Provincial People’s Hospital, The First Affiliated Hospital of Hunan Normal University, Changsha, China

^11^Department of Otolaryngology, Head and Neck Surgery, The Second Xiangya Hospital, Central South University, Changsha, Hunan, China

^12^Department of Otorhinolaryngology, Head and Neck Surgery, Shanghai Children’s Medical Center, Shanghai Jiaotong University School of Medicine, Shanghai, China

^13^Department of Otolaryngology, Head and Neck Surgery, The Children’s Hospital, Zhejiang University School of Medicine, Hangzhou, Zhejiang Province, China

^14^[Department of Otolaryngology, Head and Neck Surgery, Peking University First Hospital, Beijing, China](https://dl.acm.org/inst_page.cfm?id=60014966)

^15^Shanxi Children’s Hospital, Taiyuan, Shanxi, China

^16^Department of Otorhinolaryngology, The First Affiliated Hospital, Nanjing Medical University, Nanjing, Jiangsu, China

^17^International Centre for Allergy Research, Nanjing Medical University, Nanjing, Jiangsu, China

**Contents**

[Protocol amendments …………3](#_Toc23946538)

[Table S1. Number and percentage of subjects by age (ITT population) 5](#_Toc23946539)

[Table S2. Severity of individual nasal and ocular symptoms at baseline (*post-hoc* analysis): Summary of important protocol deviations (ITT population) 6](#_Toc23946540)

[Table S3. Summary of important protocol deviations (ITT population) 8](#_Toc23946541)

[Table S4. Mean change from baseline in daily rTOSS over first 2 weeks and 4 weeks of treatment (ITT population)…9](#_Toc23946542)

[Table S5. Mean change from baseline of intranasal finding score by anterior rhinoscopy over 2 and 4 weeks of treatment (ITT population): Primary analysis 10](#_Toc23946543)

Figure S1. Consort Diagram……………………………………………………………………………………...11

# Protocol amendments

Inclusion/exclusion criteria, screening/baseline/run-in failures, and other minor aspects of the protocol were clarified.

- Inclusion criteria: Skin prick test laboratory test aeroallergen clarified
- Exclusion criteria: Adenoid hypertrophy added
- Screening/baseline/run-in failures clarified.

# Table S1. Number and percentage of subjects by age (ITT population)

| **Age (years)** | **FFNS 55 ug once daily (n =119)** | **FFNS 110 ug once daily (n =119)** | **Placebo (n =120)** | **Total (n =358)** |
| --- | --- | --- | --- | --- |
| 2 | 3 (3%) | 8 (7%) | 1 (<1%) | 12 (3%) |
| 3 | 6 (5%) | 8 (7%) | 11 (9%) | 25 (7%) |
| 4 | 11 (9%) | 11 (9%) | 17 (14%) | 39 (11%) |
| 5 | 23 (19%) | 14 (12%) | 16 (13%) | 53 (15%) |
| 6 | 16 (13%) | 19 (16%) | 15 (13%) | 50 (14%) |
| 7 | 12 (10%) | 17 (14%) | 15 (13%) | 44 (12%) |
| 8 | 8 (7%) | 10 (8%) | 8 (7%) | 26 (7%) |
| 9 | 18 (15%) | 16 (13%) | 12 (10%) | 46 (13%) |
| 10 | 13 (11%) | 8 (7%) | 13 (11%) | 34 (9%) |
| 11 | 4 (3%) | 7 (6%) | 8 (7%) | 19 (5%) |
| 12 | 5 (4%) | 1 (<1%) | 4 (3%) | 10 (3%) |
| ≥2 to ≤6 | 59 (50%) | 60 (50%) | 60 (50%) | 179 (50%) |
| >6 to ≤12 | 60 (50%) | 59 (50%) | 60 (50%) | 179 (50%) |

ITT, intent-to-treat; FFNS, fluticasone furoate nasal spray.

# Table S2. Severity of individual nasal and ocular symptoms at baseline (*post-hoc* analysis): Summary of important protocol deviations (ITT population)

|  | FFNS 55 µg once daily (n=119) | FFNS 110 µg once daily (n=119) | Placebo (n=120) | Total (n = 358) |
| --- | --- | --- | --- | --- |
| Severity of individual nasal symptoms at baseline |  |  |  |  |
| Nasal congestion, n (%) |  |  |  |  |
| None | 0 | 0 | 0 | 0 |
| Mild | 0 | 0 | 0 | 0 |
| Moderate | 33 (28) | 32 (27) | 28 (23) | 93 (26) |
| Severe | 86 (72) | 87 (73) | 92 (77) | 265 (74) |
| Mean (SD) | 2.4 (0.38) | 2.4 (0.36) | 2.4 (0.39) | 2.4 (0.37) |
| Median | 2.3 | 2.3 | 2.4 | 2.3 |
| Min – Max | 2.0–3.0 | 2.0–3.0 | 1.5–3.0 | 1.5–3.0 |
| Runny nose, n (%) |  |  |  |  |
| None | 0 | 1 (<1) | 0 | 1 (<1) |
| Mild | 6 (5) | 3 (3) | 5 (4) | 14 (4) |
| Moderate | 64 (54) | 66 (55) | 59 (49) | 189 (53) |
| Severe | 49 (41) | 49 (41) | 56 (47) | 154 (43) |
| Mean (SD) | 2.0 (0.58) | 2.0 (0.54) | 2.1 (0.58) | 2.1 (0.57) |
| Median | 2.0 | 2.0 | 2.0 | 2.0 |
| Min – Max | 0.6–3.0 | 0.0–3.0 | 0.1–3.0 | 0.0–3.0 |
| Itchy nose, n (%) |  |  |  |  |
| None | 0 | 1 (<1) | 0 | 1 (<1) |
| Mild | 7 (6) | 10 (8) | 9 (8) | 26 (7) |
| Moderate | 66 (55) | 55 (46) | 59 (49) | 180 (50) |
| Severe | 46 (39) | 53 (45) | 52 (43) | 151 (42) |
| Mean (SD) | 2.0 (0.59) | 2.0 (0.60) | 2.1 (0.56) | 2.0 (0.58) |
| Median | 2.0 | 2.0 | 2.0 | 2.0 |
| Min – Max | 0.8–3.0 | 0.0–3.0 | 0.5–3.0 | 0.0–3.0 |
| Sneezing, n (%) |  |  |  |  |
| None | 0 | 0 | 0 | 0 |
| Mild | 5 (4) | 5 (4) | 7 (6) | 17 (5) |
| Moderate | 72 (61) | 75 (63) | 73 (61) | 220 (61) |
| Severe | 42 (35) | 39 (33) | 40 (33) | 121 (34) |
| Mean (SD) | 2.0 (0.53) | 2.0 (0.49) | 2.0 (0.53) | 2.0 (0.52) |
| Median | 2.0 | 2.0 | 2.0 | 2.0 |
| Min – Max | 1.0–3.0 | 0.1–3.0 | 0.3–3.0 | 0.1–3.0 |
| Individual ocular symptoms severity at baseline |  |  |  |  |
| Eye itching/burning, n (%) |  |  |  |  |
| None | 9 (8) | 15 (13) | 9 (8) | 33 (9) |
| Mild | 42 (35) | 39 (33) | 34 (28) | 115 (32) |
| Moderate | 52 (44) | 48 (40) | 55 (46) | 155 (43) |
| Severe | 16 (13) | 17(14) | 22 (18) | 55 (15) |
| Mean (SD) | 1.3 (0.75) | 1.3 (0.81) | 1.4 (0.72) | 1.3 (0.76) |
| Median | 1.1 | 1.1 | 1.4 | 1.3 |
| Min – Max | 0.0–3.0 | 0.0–3.0 | 0.0–3.0 | 0.0–3.0 |
| Eye tearing/watering, n (%) |  |  |  |  |
| None | 25 (21) | 27 (23) | 20 (17) | 72 (20) |
| Mild | 54 (45) | 47 (39) | 40 (33) | 141 (39) |
| Moderate | 36 (30) | 41 (34) | 55 (46) | 132 (37) |
| Severe | 4 (3) | 4 (3) | 5 (4) | 13 (4) |
| Mean (SD) | 0.8 (0.66) | 0.9 (0.71) | 1.0 (0.69) | 0.9 (0.69) |
| Median | 0.9 | 1.0 | 1.1 | 1.0 |
| Min – Max | 0.0–2.5 | 0.0–2.5 | 0.0–2.5 | 0.0–2.5 |
| Eye redness, n (%) |  |  |  |  |
| None | 25 (21) | 28 (24) | 25 (21) | 78 (22) |
| Mild | 56 (47) | 50 (42) | 43 (36) | 149 (42) |
| Moderate | 32 (27) | 35 (29) | 41 (34) | 108 (30) |
| Severe | 6 (5) | 6 (5) | 11 (9) | 23 (6) |
| Mean (SD) | 0.8 (0.74) | 0.9 (0.77) | 1.0 (0.76) | 0.9 (0.76) |
| Median | 0.8 | 1.0 | 1.0 | 1.0 |
| Min – Max | 0.0–3.0 | 0.0–3.0 | 0.0–3.0 | 0.0–3.0 |

ITT, intent to treat; FFNS, fluticasone furoate nasal spray; SD, standard deviation.

# Table S3. Summary of important protocol deviations (ITT population)

| **Protocol deviation, n (%)** | **FFNS 55 µg  once daily (n=119)** | **FFNS 110 µg  once daily (n=119)** | **Placebo (n=120)** | **Total (n=358)** |
| --- | --- | --- | --- | --- |
| Any deviation | 26 (22) | 37 (31) | 38 (32) | 101 (28) |
| Assessment or time point completion | 0 | 0 | 2 (2) | 2 (<1) |
| Other assessment or time point window | 0 | 0 | 2 (2) | 2 (<1) |
| Eligibility criteria not met | 5 (4) | 6 (5) | 10 (8) | 21 (6) |
| Excluded medication | 11 (9) | 17 (14) | 13 (11) | 41 (11) |
| Medication excluded by the protocol was administered | 11 (9) | 17 (14) | 13 (11) | 41 (11) |
| Informed consent | 0 | 0 | 1 (<1) | 1 (<1) |
| Wrong informed consent/assent version signed | 0 | 0 | 1 (<1) | 1 (<1) |
| Not withdrawn after developing withdrawal criteria | 6 (5) | 7 (6) | 6 (5) | 19 (5) |
| Not discontinued from study treatment | 6 (5) | 7 (6) | 6 (5) | 19 (5) |
| Study procedures | 0 | 8 (7) | 6 (5) | 14 (4) |
| Diary procedures | 0 | 1 (<1) | 0 | 1 (<1) |
| Other deviation from study procedures | 0 | 1 (<1) | 0 | 1 (<1) |
| Randomization procedures (e.g., patient assigned to wrong stratum, patient randomized out of order) | 0 | 6 (5) | 6 (5) | 12 (3) |
| Wrong study treatment/administration/dose | 9 (8) | 10 (8) | 11 (9) | 30 (8) |
| Study treatment not administered per protocol | 9 (8) | 10 (8) | 11 (9) | 30 (8) |

Patients could have more than 1 important protocol deviation.

FFNS, fluticasone furoate nasal spray; ITT, intent-to-treat.

#

# Table S4. Mean change from baseline in daily rTOSS over first 2 weeks and 4 weeks of treatment (ITT population)

| Daily rTOSS | All patients^‡^ | | | | ≥2 to ≤6 years^§^ | | | | >6 to ≤12 years^§^ | | | |
| --- | --- | --- | --- | --- | --- | --- | --- | --- | --- | --- | --- | --- |
|  | FFNS 55 µg once daily (n = 119) | FFNS 110 µg once daily (n = 119) | FFNS 55/110 µg once daily (n = 238) | Placebo (n = 120) | FFNS 55 µg once daily (n = 59) | FFNS 110 µg once daily (n = 60) | FFNS 55/110 µg once daily (n = 119) | Placebo (n = 60) | FFNS 55 µg once daily (n = 60) | FFNS 110 µg once daily (n = 59) | FFNS 55/110 µg once daily (n = 119) | Placebo (n = 60) |
| Baseline mean (SD) | 2.9 (1.92) | 3.1 (2.09) | 3.0 (2.00) | 3.4 (1.94) |  |  |  |  |  |  |  |  |
| Week 1–2 mean (SD) | 1.8 (1.60) | 1.7 (1.72) | 1.7 (1.66) | 2.1 (1.52) |  |  |  |  |  |  |  |  |
| Week 1–4 mean (SD) | 1.5 (1.46) | 1.4 (1.55) | 1.5 (1.50) | 1.9 (1.37) |  |  |  |  |  |  |  |  |
| Week 1–2 change from baseline |  |  |  |  |  |  |  |  |  |  |  |  |
| Mean change (SD) | −1.2 (1.27) | −1.4 (1.43) | −1.3 (1.35) | −1.2 (1.35) |  |  |  |  |  |  |  |  |
| LS mean change (SE)* | −1.24 (0.10) | −1.40 (0.10) | −1.32(0.07) | −1.15 (0.10) | −1.26 (0.14) | −1.38 (0.14) | −1.32 (0.10) | −1.22 (0.14) | −1.21 (0.14) | −1.42 (0.15) | −1.31 (0.10) | −1.09 (0.14) |
| LS mean difference* | −0.1 | −0.25 | −0.17 |  | −0.04 | −0.16 | −0.10 |  | −0.13 | −0.33 | −0.23 |  |
| *P*-value vs. placebo* | 0.503 | 0.078 | 0.16 |  | 0.837 | 0.408 | 0.551 |  | 0.533 | 0.111 | 0.200 |  |
| 95% CI* | (−0.38, 0.18) | (−0.53, 0.03) | (−0.42, 0.07) |  | (−0.44, 0.35) | (−0.56, 0.23) | (−0.44, 0.24) |  | (−0.53, 0.28) | (−0.74, 0.08) | (−0.58, 0.12) |  |
| *P*-value vs. FFNS 55 µg |  |  |  |  |  | 0.538 |  |  |  | 0.325 |  |  |
| 95% CI† |  |  |  |  |  | (−0.52, 0.27) |  |  |  | (−0.61, 0.20) |  |  |
| Week 1–4 change from baseline |  |  |  |  |  |  |  |  |  |  |  |  |
| Mean change (SD) | −1.4 (1.39) | −1.7 (1.52) | −1.6 (1.46) | −1.5 (1.46) |  |  |  |  |  |  |  |  |
| LS mean change (SE)* | −1.52 (0.10) | −1.70 (0.10) | −1.61 (0.07) | −1.41 (0.10) | −1.56 (0.14) | −1.71 (0.14) | −1.63 (0.10) | −1.51 (0.14) | −1.45 (0.14) | −1.70 (0.14) | −1.58 (0.10) | −1.31 (0.14) |
| LS mean difference* | −0.11 | −0.3 | −0.2 |  | −0.05 | −0.19 | −0.12 |  | −0.14 | −0.39 | −0.26 |  |
| *P*-value vs. placebo* | 0.442 | 0.035 | 0.096 |  | 0.793 | 0.317 | 0.466 |  | 0.489 | 0.054 | 0.130 |  |
| 95% CI* | (−0.38, 0.17) | (−0.57, −0.02) | (−0.44, 0.04) |  | (−0.44, 0.33) | (−0.57, 0.19) | (−0.45, 0.21) |  | (−0.53, 0.26) | (−0.79, 0.01) | (−0.61, 0.08) |  |
| *P*-value vs. FFNS 55 µg |  |  |  |  |  | 0.464 |  |  |  | 0.211 |  |  |
| 95% CI^†^ |  |  |  |  |  | (−0.53, 0.24) |  |  |  | (−0.65, 0.14) |  |  |

*The baseline period for diary card endpoints was defined as 4 days before randomization, including the morning symptom assessment on the randomization (*i.e*., treatment initiation) date.

^†^Based on ANCOVA adjusting for baseline daily rTNSS, classification of allergic rhinitis (intermittent or persistent), age (as continuous variable), sex, and treatment.

^‡^Primary analysis data.

^§^*Post-hoc* analysis data.

CI, confidence interval; FFNS, fluticasone furoate nasal spray; ITT, intent-to-treat; LS, least-squares; LS mean difference, LS mean change in active treatment minus LS mean change in placebo; rTNSS, reflective total nasal symptom scores; rTOSS, reflective total ocular symptom scores; SD, standard deviation; SE, standard error.

# Table S5. Mean change from baseline of intranasal finding score by anterior rhinoscopy over 2 and 4 weeks of treatment (ITT population): Primary analysis

| Intranasal finding score by anterior rhinoscopy | FFNS 55 µg  once daily (n = 119) | FFNS 110 µg  once daily (n = 119) | FFNS 55/110 µg once daily (n = 120) | Placebo (n = 358) |
| --- | --- | --- | --- | --- |
| Baseline,* n | 119 | 119 | 238 | 120 |
| Mean (SD) | 9.7 (1.61) | 9.6 (1.67) | 9.7 (1.64) | 9.6 (1.60) |
| Week 2, n | 114 | 119 | 233 | 114 |
| Mean (SD) | 5.2 (2.83) | 5.4 (2.80) | 5.3 (2.81) | 7.4 (2.76) |
| Week 4, n | 111 | 111 | 222 | 106 |
| Mean (SD) | 4.0 (2.85) | 4.2 (2.63) | 4.1 (2.74) | 6.5 (3.11) |
| Change from baseline |  |  |  |  |
| Week 2, n | 114 | 119 | 233 | 114 |
| Mean change (SD) | −4.5 (2.82) | −4.3 (2.92) | −4.4 (2.87) | −2.3 (2.51) |
| LS mean change (SE)^†^ | −4.47 (0.25) | −4.30 (0.24) | −4.38 (0.17) | −2.32 (0.25) |
| LS mean difference^†^ | −2.15 | −1.98 | −2.06 |  |
| *P*-value vs. placebo^†^ | <0.001 | <0.001 | <0.001 |  |
| 95% CI^†^ | (−2.84, −1.46) | (−2.66, −1.29) | (−2.66, −1.47) |  |
| Week 4, n | 111 | 111 | 222 | 106 |
| Mean change (SD) | −5.7 (2.91) | −5.5 (2.94) | −5.6 (2.92) | −3.2 (3.22) |
| LS mean change (SE)^†^ | −5.68 (0.27) | −5.48 (0.27) | −5.58 (0.19) | −3.21 (0.27) |
| LS mean difference^†^ | −2.48 | −2.27 | −2.38 |  |
| *P*-value vs. placebo^†^ | <0.001 | <0.001 | <0.001 |  |
| 95% CI^†^ | (−3.23, −1.73) | (−3.03, −1.52) | (−3.03, −1.72) |  |

*The baseline period for diary card endpoints was defined as 4 days before randomization, including the morning symptom assessment on the randomization (i.e., treatment initiation) date.

^†^LS mean, SE, CI and *P*-value are from an ANCOVA model with treatment, baseline value, sex, age and type of allergic rhinitis.
CI, confidence interval; FFNS, fluticasone furoate nasal spray; IT, intent-to-treat; LS, least-square; LS mean difference, LS mean change in active treatment minus LS mean change in placebo; SD, standard deviation; SE, standard error.


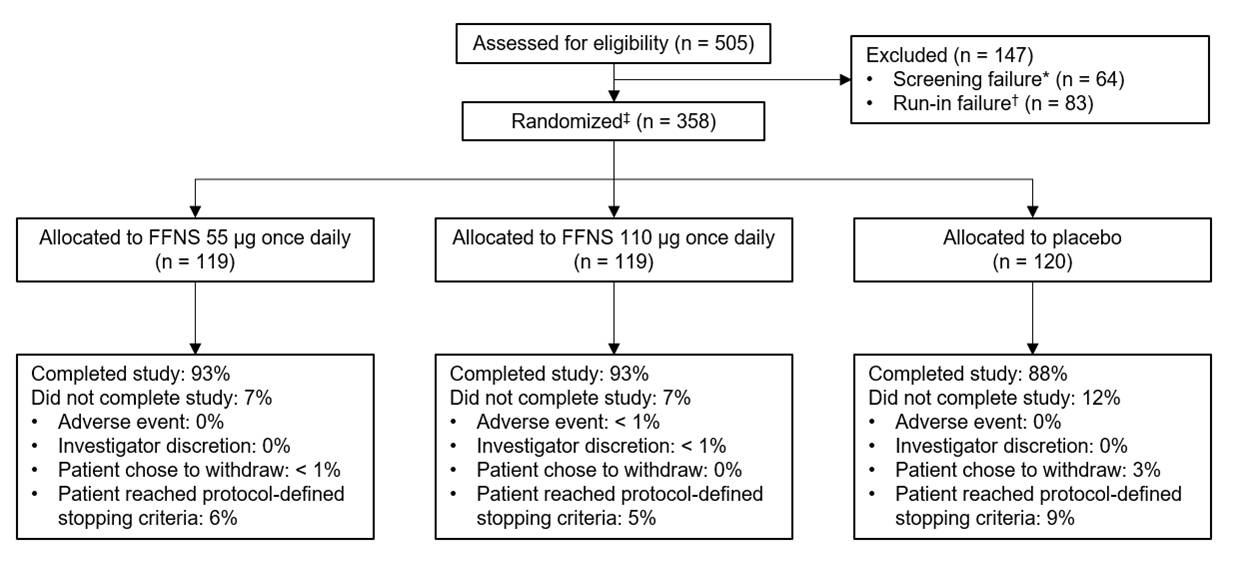


## Fig. S1. Consort diagram. FFNS, fluticasone furoate nasal spray; *Screening failure: patients who failed to meet eligibility requirements at Visit 1. ^†^Run-in failure: patients who failed to meet eligibility requirements at Visit 2. ^‡^Two patients who failed screening were randomized in error but were not included in the analysis. Protocol-defined stopping criteria: a subject could voluntarily discontinue participation in this study at any time. The investigator could also, at his or her discretion, discontinue a subject from this study if it was detrimental for them to continue to participate.
